# Efficacy and safety of arbidol (umifenovir) in patients with COVID‐19: A systematic review and meta‐analysis

**DOI:** 10.1002/iid3.502

**Published:** 2021-08-04

**Authors:** Behnam Amani, Bahman Amani, Sara Zareei, Mahsa Zareei

**Affiliations:** ^1^ Department of Health Management and Economics, School of Public Health Tehran University of Medical Sciences Tehran Iran; ^2^ Health Management and Economics Research Center, Health Management Research Institute Iran University of Medical Sciences Tehran Iran; ^3^ Department of Cell & Molecular Biology, Faculty of Biological Sciences Kharazmi University Tehran Iran; ^4^ Department of Health Services Management, School of Health Management and Information Sciences Iran University of Medical Sciences Tehran Iran

**Keywords:** 2019 novel coronavirus infection, 2019‐nCoV infection, arbidol, coronavirus, novel coronavirus, umifenovir

## Abstract

**Objective:**

To provide the latest evidence for the efficacy and safety of arbidol (umifenovir) in COVID‐19 treatment.

**Methods:**

A literature systematic search was carried out in PubMed, Cochrane Library, Embase, and medRxiv up to May 2021. The Cochrane risk of bias tool and Newcastle–Ottawa scale were used to assess the quality of included studies. Meta‐analysis was performed using RevMan 5.3.

**Results:**

Sixteen studies were met the inclusion criteria. No significant difference was observed between arbidol and non‐antiviral treatment groups neither for primary outcomes, including the negative rate of PCR (NR‐PCR) on Day 7 (risk ratio [RR]: 0.94; 95% confidence interval (CI): 0.78–1.14) and Day 14 (RR: 1.10; 95% CI: 0.96–1.25), and PCR negative conversion time (PCR‐NCT; mean difference [MD]: 0.74; 95% CI: −0.87 to 2.34), nor secondary outcomes (*p *> .05). However, arbidol was associated with higher adverse events (RR: 2.24; 95% CI: 1.06–4.73). Compared with lopinavir/ritonavir, arbidol showed better efficacy for primary outcomes (*p *< .05). Adding arbidol to lopinavir/ritonavir also led to better efficacy in terms of NR‐PCR on Day 7 and PCR‐NCT (*p *< .05). There was no significant difference between arbidol and chloroquine in primary outcomes (*p *> .05). No remarkable therapeutic effect was observed between arbidol and other agents (*p *> .05).

**Conclusion:**

The present meta‐analysis showed no significant benefit of using arbidol compared with non‐antiviral treatment or other therapeutic agents against COVID‐19 disease. High‐quality studies are needed to establish the efficacy and safety of arbidol for COVID‐19.

## Graphical abstract

The evidence on the antivirus effect of Arbidol against COVID‐19 is controversial. The present meta‐analysis showed no significant benefit of using arbidol compared with non‐antiviral treatment or other therapeutic agents against COVID‐19 disease

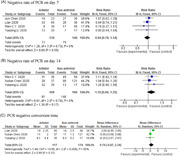

## 1 INTRODUCTION

Severe acute respiratory syndrome coronavirus 2 (SARS‐CoV‐2), the causative agent of coronavirus disease 2019 (COVID‐19), has rapidly spread throughout the world leading to a pandemic.^1^, ^2^, ^3^ Up until now, some antiviral drugs have been proposed as promising therapeutic agents against SARS‐CoV‐2 infection including interferon,^4^ lopinavir/ritonavir,^5^ chloroquine,^6^ remdesivir,^7^ and arbidol.^8^

Arbidol (umifenovir) is an oral antiviral drug^9^ that was approved for prophylaxis in Russia and China several decades ago and used in the treatment of influenza A and B as well as other respiratory viral infections.^10^ In addition to Arbidol's antiviral and anti‐inflammatory activities against various types of influenza viruses,^11^, ^12^ especially H1N1,^13^ its broad‐spectrum antiviral activities against other viruses, such as Zika,^14^ Ebola,^15^ hepatitis B and C,^16^, ^17^ rhinovirus,^18^ respiratory syncytial virus,^18^, ^19^ coxsackie,^18^, ^20^ chikungunya,^21^ and adenovirus^18^ are shown in vitro and in vivo.

Regarding the SARS‐CoV‐2 infection, the antivirus effect of arbidol against SARS‐CoV‐2 has yet been controversial. On the one hand, the efficacy of arbidol was shown in vitro^22^, ^23^ which seems to have inhibited the infection more efficiently among other WHO‐approved anti‐influenza drugs including baloxavir, laninamivir, oseltamivir, peramivir, zanamivir^23^ by blocking the trimerization of the spike glycoprotein.^22^ Also, some studies suggested its beneficial effects either in monotherapy or combination therapy with other agents against COVID‐19.^5^, ^24^, ^25^, ^26^ On the other hand, there exist other studies which have found no benefit of using arbidol in COVID‐19 patients ^27^, ^28^ suggesting an urgent need to reach a conclusive decision on this matter. The present systematic review and meta‐analysis aim to provide the latest evidence on arbidol's efficacy and safety compared with other therapeutic agents in COVID‐19 treatment.

## 2 METHODS

We have registered the protocol of this systematic review and meta‐analysis with the registry number CRD42020207821 and used the Preferred Reporting Items for Systematic Reviews and Meta‐Analyses (PRISMA) checklist.^29^

### 2.1 Literature search strategy

We conducted a systematic search in the leading bibliographic databases, including PubMed, the Cochrane Library, and Embase for the relevant records up to May 2021. We also searched in medRxiv, Google Scholar, and clinical registry databases, including ClinicalTrials.gov, the European Union Clinical Trials Register, and the Chinese Clinical Trial Registry for additional relevant documents. Finally, the reference lists of the included studies and review articles were screened and the search was limited to the articles the abstract or full text of which were in English. Search terms included 2019‐nCoV, SARS‐CoV‐2, COVID‐19, arbidol, and umifenovir. The following terms were used to explore PubMed: ((((((((Coronavirus[Title/Abstract]) OR (Coronavirus[MeSH Terms])) OR (COVID‐19[Title/Abstract])) OR (SARS‐CoV‐2[Title/Abstract])) OR (COVID‐19[MeSH Terms])) OR (SARS‐CoV‐2[MeSH Terms])) OR (2019 novel coronavirus infection[Title/Abstract])) OR (2019‐nCoV infection[Title/Abstract])) AND ((Umifenovir[Title/Abstract]) OR (Arbidol[Title/Abstract])).

### 2.2 Study selection

Two authors (Behnam Amani and Mahsa Zareei) independently screened the identified records based on inclusion and exclusion criteria. Disagreements between the authors were resolved by discussion among authors. The studies were included based on the following criteria: (1) patients with laboratory‐confirmed positive COVID‐19 test; (2) arbidol as monotherapy or in combination with other therapeutic agents; (3) any therapeutic intervention as a comparison (4); efficacy and safety outcomes of interest. The primary efficacy outcomes were the negative rate of PCR (polymerase chain reaction) and PCR negative conversion time and the secondary efficacy outcomes included the rate of improvement on chest CT, rate of cough alleviation, length of hospital stay, and disease progression. The safety outcome was the incidence of adverse events reported in patients; and (5) clinical trials or observational studies. The exclusion criteria were the studies conducted on animal models, case reports, case series, letters to editors, and editorials.

### 2.3 Data extraction and quality assessment

We used the Cochrane collaboration tool to assess the risk of bias of randomized clinical trials.^30^ Quality assessment of observational studies was conducted using the Newcastle–Ottawa scale (NOS).^31^ We extracted data using the same data extraction form. The extracted data included (1) study characteristics (author, year, setting, and design); (2) patient's characteristics (sample size, sex, and age); (3) intervention and comparison (sample size); and (4) efficacy and safety outcomes. All steps were performed independently by two authors (Behnam Amani and Mahsa Zareei).

### 2.4 Evidence synthesis

We performed a meta‐analysis using RevMan software, version 5.3. The mean difference (MD) with a 95% confidence interval (CI) was used for continuous variables and a risk ratio (RR) with 95% CI for dichotomous variables. The statistical heterogeneity was evaluated using the *I* ^2^ and Chi^2^ tests. The random‐effects model was used for studies with *I* ^2^ > 50% or *p* < .1. Otherwise, we used the fixed‐effect model.

## 3 RESULTS

### 3.1 The characteristics of studies

**Figure 1:**
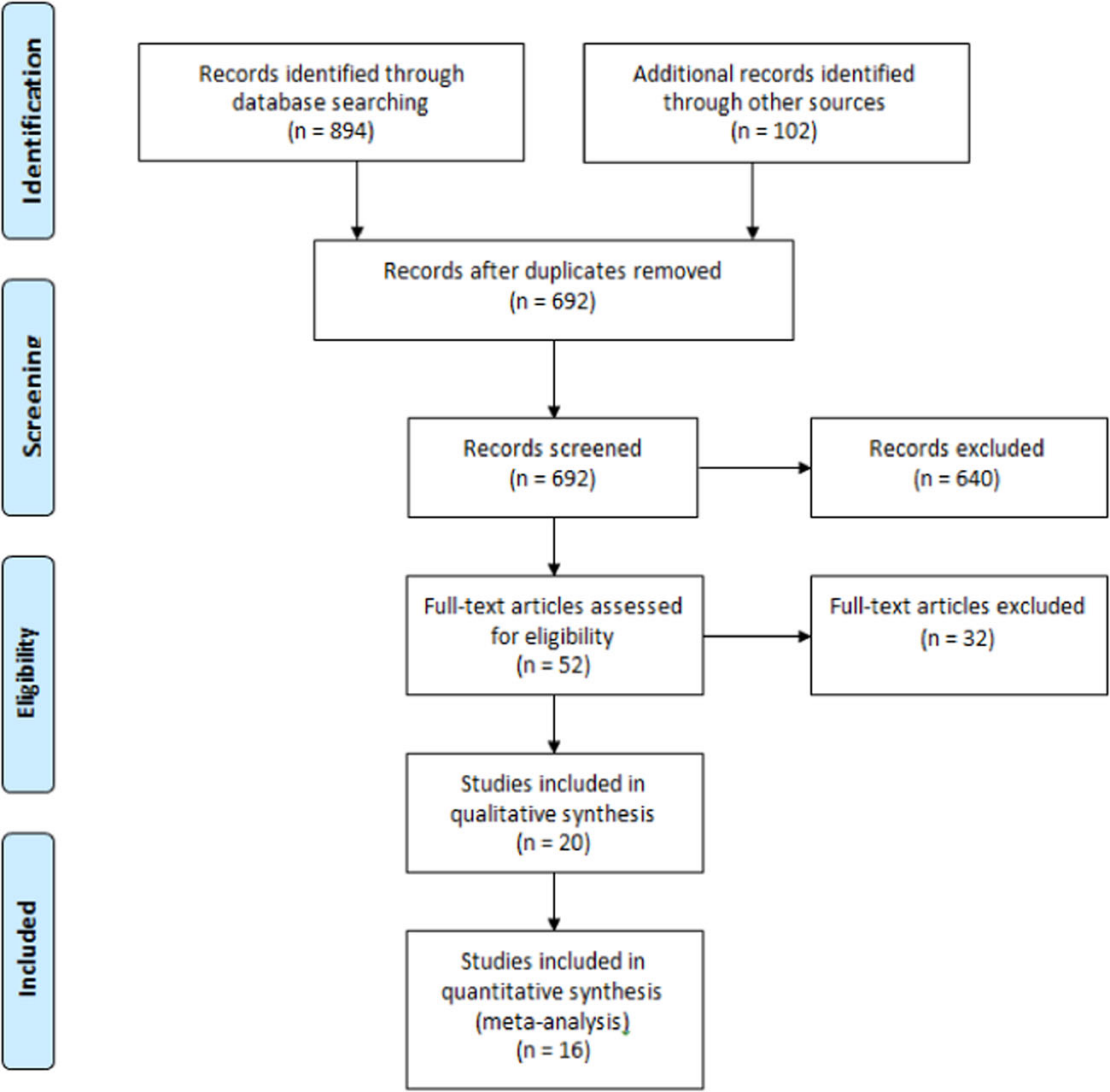
Study flow diagram

**Figure 2:**
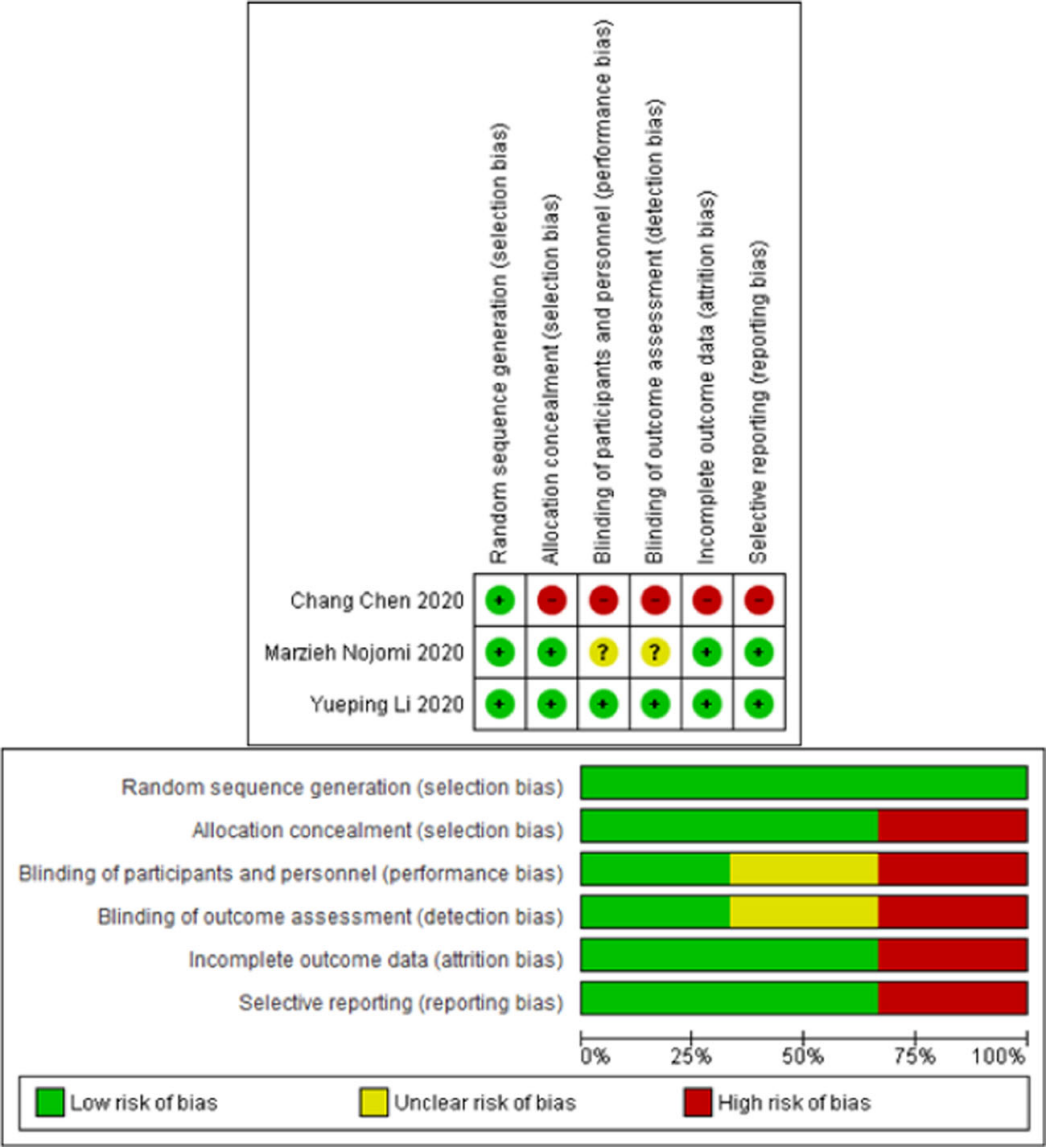
Risk of bias in the selected studies

Figure 1 shows the literature search flow, removal of duplicates, and the screening based on title, abstract, and full text. As a result, 52 full‐text articles were reviewed and sixteen studies^24^, ^32^, ^33^, ^34^, ^35^, ^36^, ^37^, ^38^, ^39^, ^40^, ^41^, ^42^, ^43^, ^44^, ^45^, ^46^ entered the final analysis. The characteristics of the studies included in the systematic review are presented in Table 1. Assessment of the risk of bias using the Cochrane collaboration tool is presented in Figure 2.

**Table 1:** Characteristics of included studies

Study, year	Country	Design	Age (mean)	*N* (M/F)	Intervention (*n*)	Comparison (*n*)	NOS
Chang Chen 2020,^47^	China	RCT	NA	236 (110/126)	Arbidol (120)	Favipiravir (116)	RoB 2
Huang 2020,^24^	China	R	NA	27 (12/15)	Arbidol (11)	LPV/r (6), CQ (10)	7
Lisi Deng 2020,^33^	China	R	44.5	33 (17/16)	Arbidol + LPV/r (16)	LPV/r (17)	8
Ping Xu 2020,^42^	China	R	51.9	141 (74/67)	Arbidol + IFN (71)	IFN (70)	6
Qibin Liu 2020,^25^	China	R	59.5	504 (259/245)	Arbidol (257)	Os (66), LPV/r (259)	6
Qiong Zhou 2020,^44^	China	R	NA	77 (31/46)	Arbidol (24)	Arbidol + IFN (46), IFN (7)	7
Wenyu Chen 2020,^48^	China	RCT	NA	62 (34/28)	Arbidol + control (42)	Control (20)	RoB 2
Kaijin Xu 2020,^26^	China	R	NA	111 (47/64)	Arbidol + ER (49)	ER (62)	7
Xiu Lan 2020,^35^	China	R	55.8	73 (37/36)	Arbidol + LPV/r (39)	LPV/r (34)	7
Jun Chen 2020,^34^	China	R	48	134 (69/65)	Arbidol (34)	LPV/r (52), non‐antiviral (48)	5
Xudan Chen 2020,^32^	China	R	48	284 (131/153)	Arbidol (37)	Control (121), LPV/r (60), arbidol + LPV/r (16), CQ (17), Os (13), Other (16)	9
Yaya Zhou 2020,^45^	China	R	55.5	238 (102/136)	Arbidol (82)	Arbidol + IFN (139)	7
Yueping Li 2020,^37^	China	RCT	49.4	86 (40/46)	Arbidol (35)	LPV/r (34), control (17)	RoB 2
Zhu 2020,^46^	China	R	39.8	50 (26/24)	Arbidol (16)	LPV/r (34)	7
Wen 2020,^41^	China	R	49.9	178 (81/97)	Arbidol (36)	LPV/r (59), control (58), arbidol + LPV/r (25)	7
Ming Li 2021,^36^	China	R	NA	62 (24/38)	Arbidol (42)	CQ (20)	7
Jie 2021,^49^	China	R	65	252 (106/146)	Arbidol (228)	No arbidol (24)	8
Ruan 2021,^50^	China	R	64	331 (160/171)	Arbidol (273)	Non‐antiviral (58)	8
Ghaderkhani 2021,^51^	Iran	RCT	NA	53 (32/21)	HCQ + arbidol (28)	HCQ (25)	RoB 2
Nojomi 2020,^40^	Iran	RCT	56.4	100 (60/40)	Arbidol (50)	LPV/r (50)	RoB 2
Lian 2020,^38^	China	R	60	81 (45/36)	Arbidol (45)	Control (36)	8
Liu 2021,^39^	China	R	54.8	108 (47/61)	Arbidol (40)	Arbidol + LHQW (68)	8
Jing Chen 2020,^52^	China	R	NA	200 (130/70)	Arbidol + SFJDC (100)	Arbidol (100)	8
Fang 2020,^53^	China	R	61.5	162 (87/75)	Arbidol + LHQW (113)	LHQW (49)	8
Ping 2020,^43^	China	R	NA	295 (171/124)	Arbidol (148)	LHQW + arbidol (147)	8
Xiang‐Kun 2020,^54^	China	R	NA	70 (41/29)	Arbidol (30)	SFJD + arbidol (40)	9

Abbreviations: CQ, chloroquine; ER, empirical regimens; F, female; HCQ, hydroxychloroquine; IFN, Interferon; LPV/r, lopinavir/ritonavir; LHQW: Lianhuaqingwen; M, male; N, number; NA, not acquired; Os, oseltamivir; R, retrospective; RCT, randomized clinical trial; RoB, risk of bias; SFJD, Shufeng Jiedu.

### 3.2 Comparisons

#### 3.2.1 Arbidol versus non‐antiviral treatment

**Figure 3:**
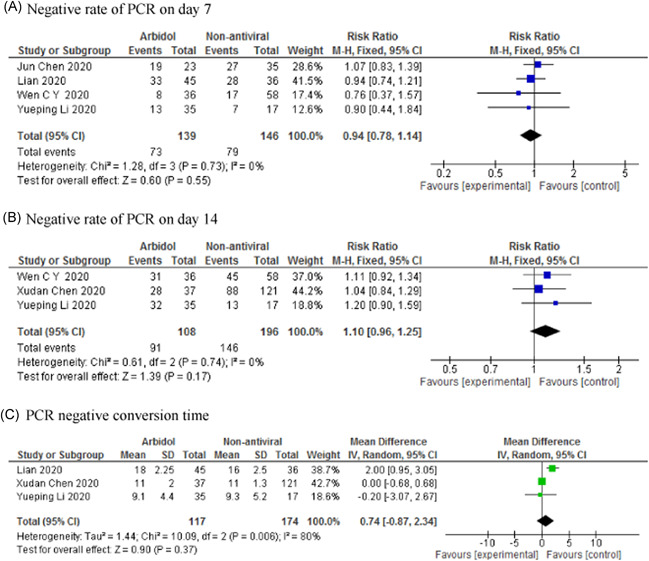
Forest plot of arbidol versus non‐antiviral for outcomes of negative rate of PCR on Day 7 (A), negative rate of PCR on Day 14 (B), and PCR negative conversion time (C)

**Figure 4:**
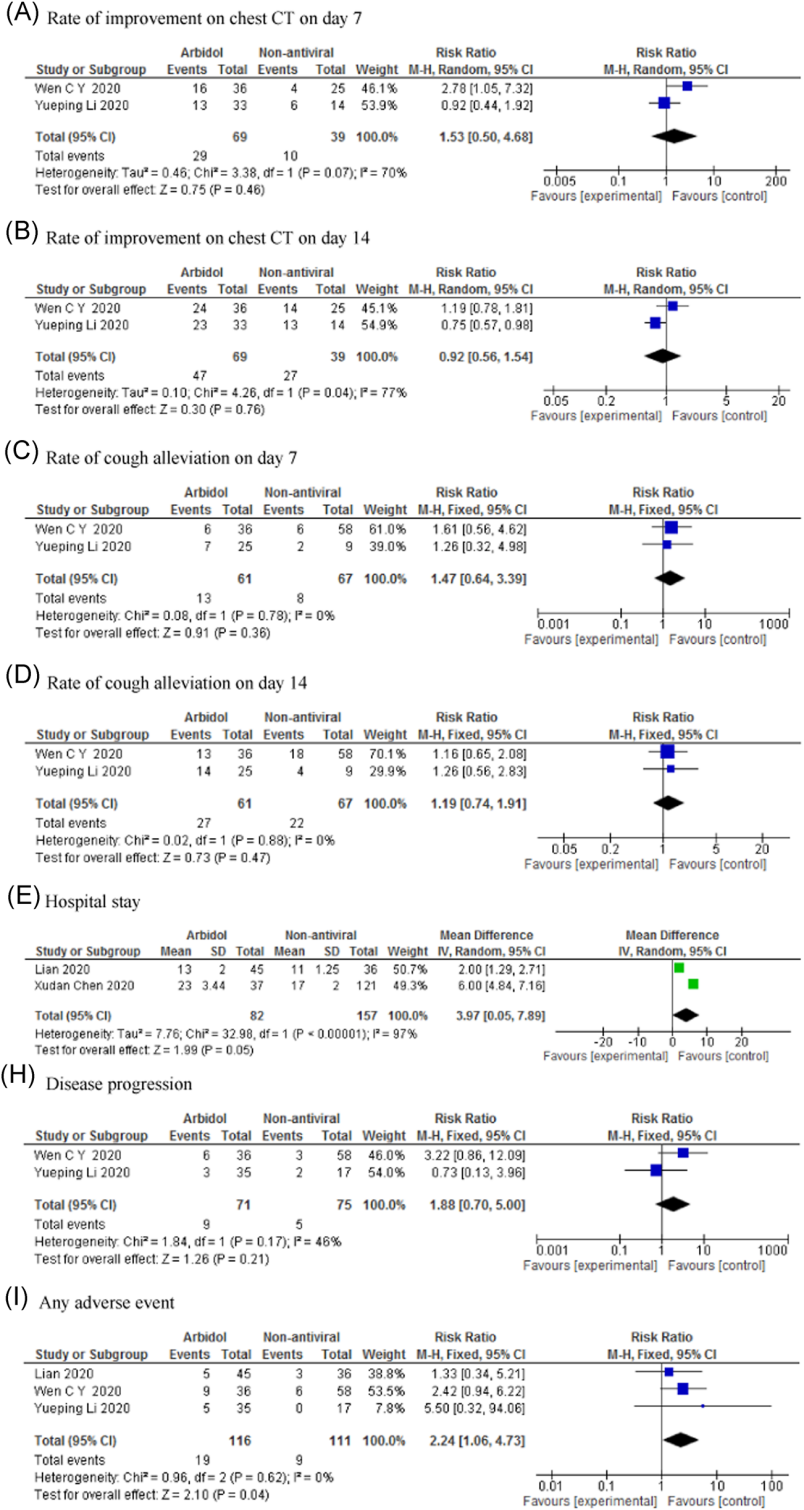
Forest plot of arbidol versus non‐antiviral for outcomes of rate of improvement on chest CT on Day 7 (A), rate of improvement on chest CT on Day 14 (B), rate of cough alleviation on Day 7 (C), rate of cough alleviation on Day 14 (D), hospital stay (E), disease progression (H), and any adverse event (I)

The result of meta‐analysis showed that there was no significant difference between arbidol and non‐antiviral groups in terms of negative rate of PCR on Day 7 (RR: 0.94; 95% CI: 0.78–1.14; *p *= .55) and Day 14 (RR: 1.10; 95% CI: 0.96–1.25; *p *= .17), PCR negative conversion time (MD: 0.74; 95% CI: −0.87 to 2.34; *p *= .37) (Figure 3), rate of improvement on chest CT on Day 7 (RR: 1.53; 95% CI: 0.50–4.68; *p *= .46) and Day 14 (RR: 0.92; 95% CI: 0.56–1.54; *p *= .76), rate of cough alleviation on Day 7 (RR: 1.47; 95% CI: 0.64–3.39; *p *= .36) and Day 14 (RR: 1.19; 95% CI: 0.74–1.91; *p *= .47), hospital stay (MD: 3.97; 95% CI: 0.05–7.89; *p *= .05), and disease progression (RR: 1.88; 95% CI: 0.70–5.00; *p *= .21; Figure 4). Arbidol was associated with higher adverse events (RR: 2.24; 95% CI: 1.06–4.73; *p *= .04; Figure 4).

#### 3.2.2 Arbidol versus favipiravir

Only one study^47^ compared arbidol with favipiravir. The result showed no significant difference between arbidol and favipiravir groups in the clinical recovery rate. However, favipiravir was associated with better efficacy in relieving pyrexia and cough. The frequencies of drug‐related adverse events for arbidol and favipiravir were 23.33% and 31.9%, respectively.

#### 3.2.3 Arbidol versus chloroquine

There was no significant difference between arbidol and chloroquine in terms of negative rate of PCR on Day 14 (RR: 1.27; 95% CI: 0.64–2.51; *p *= .50) and PCR negative conversion time (MD: 0.69; 95% CI: −3.72 to 5.10; *p *= .76; Table 2). However, the length of hospital stay in patients taking chloroquine was significantly shorter than patients taking arbidol (MD: 4.59; 95% CI: 0.58–8.60; *p *= .02; Table 2).

**Table 2:** The pooled estimate of arbidol versus other therapeutic agents and sensitivity analysis

Analysis	No. of studies	Participants	Pooled estimate (95% CI)	*p*	Heterogeneity		
					Ch^2^	*p*	*I* ^2^
*Sensitivity analysis*							
Arbidol versus non‐antiviral							
Negative rate of PCR	4	405	1.21 (1.06–1.38)	.005	5.79	.12	48%
*Arbidol versus chloroquine*							
Negative rate of PCR on Day 14	3	137	1.27 (0.64–2.51)	.50	12.54	.002	84%
PCR negative conversion time	2	75	0.69 (−3.72 to 5.10)	.76	14.71	.0001	93%
Hospital stay	2	75	4.59 (0.58–8.60)	.02	8.44	.004	88%
*Arbidol versus LPV/r*							
Negative rate of PCR on Day 7	4	276	1.35 (1.03–1.76)	.03	4.18	.24	28%
Negative rate of PCR on Day 14	5	328	1.47 (1.06–2.04)	.02	24.07	<.0001	83%
PCR negative conversion time	5	328	−2.28 (−3.83 to −0.72)	.004	21.91	.0002	82%
Hospital stay	3	214	−1.87 (−8.01 to 4.27)	.55	50.39	<.00001	96%
Rate of improvement on chest CT on Day 7	2	156	1.14 (0.77–1.69)	.50	0.29	0.59	0%
Rate of improvement on chest CT on Day 14	2	156	0.99 (0.80–1.23)	.92	0.24	0.62	0%
Disease progress	2	164	1.08 (0.13–9.29)	.94	5.64	0.02	82%
Rate of cough alleviation on Day 7	2	141	1.61 (0.21–12.22)	.64	5.48	0.02	82%
Rate of cough alleviation on Day 14	2	141	0.81 (0.58–1.15)	.24	0.32	0.57	0%
Adverse events	5	367	0.44 (0.28–0.68)	.0002	2.70	0.61	0%
*Arbidol + LPV/r versus LPV/r*							
Negative rate of PCR on Day 7	2	117	2.06 (1.13–3.76)	.02	0.01	0.91	0%
Negative rate of PCR on Day 14	3	193	0.99 (0.55–1.80)	.99	9.44	0.009	79%
PCR negative conversion time	3	229	2.21 (−0.13 to 4.54)	.06	6.61	0.04	70%
Hospital stay	2	145	1.51 (−3.94 to 6.97)	.59	6.46	0.01	85%
Rate of imrovement on chest CT on Day 7	2	117	1.05 (0.20–5.50)	.96	6.99	0.008	86%
*Arbidol versus arbidol + IFN*							
PCR negative conversion time	2	291	−0.99 (−16.67 to 14.69)	.90	715.70	<.00001	100%
Arbidol + IFN versus IFN							
PCR negative conversion time	2	194	2.31 (−7.78 to 12.40)	.65	28.11	<.00001	96%
Arbidol + LHQW versus arbidol							
Rate of improvement on chest CT	2	403	1.27 (0.88–1.85)	.20	2.91	0.09	66%

Abbreviations: CI, confidence interval; IFN, interferon; LHQW, Lianhuqingwen; LPV/r, lopinavir/ritonavir; P, p‐value; PCR, polymerase chain reaction.

#### 3.2.4 Arbidol versus oseltamivir

Chen et al.^27^ found that the clearance rate of arbidol and oseltamivir during 14 days were 75.7% and 61.5%, respectively. The median length of hospital stay in both groups was similar. The result of another study^25^ showed that arbidol was more effective than oseltamivir in reducing mortality. Also, arbidol was more effective in the reduction of lesion size (46.43% vs. 41.18%).

#### 3.2.5 Arbidol versus lopinavir/ritonavir

Arbidol showed better efficacy compared to lopinavir/ritonavir in terms of negative rate of PCR on Day 7 (RR: 1.35; 95% CI: 1.03–1.76; *p *= .03) and Day 14 (RR: 1.47; 95% CI: 1.06–2.04; *p *= .02), as well as PCR negative conversion time (MD: −2.28; 95% CI: −3.83 to − 0.72; *p *= .004; Table 2). However, there was no significant difference between two drugs in terms of rate of improvement on chest CT on Day 7 (RR: 1.14; 95% CI: 0.77–1.69; *p *= .50) and Day 14 (RR: 0.99; 95% CI: 0.80–1.23; *p *= .92), rate of cough alleviation on Day 7 (RR: 1.61; 95% CI: 0.21–12.22; *p *= .64) and Day 14 (RR: 0.81; 95% CI: 0.58–1.15; *p *= .24), hospital stay (MD: −1.87; 95% CI: −8.01 to 4.27; *p *= .55), and disease progression (RR: 1.08; 95% CI: 0.13–9.29; *p *= .94; Table 2). Compared with lopinavir/ritonavir, arbidol had fewer adverse events (RR: 0.44; 95% CI: 0.28–0.68; *p *= .0002; Table 2).

#### 3.2.6 Arbidol plus lopinavir/ritonavir versus lopinavir/ritonavir

Arbidol combined with lopinavir/ritonavir versus exclusive administration of lopinavir/ritonavir was associated with higher negative rate of PCR on Day 7 (RR: 2.06; 95% CI: 1.13–3.76; *p *= .02; Table 2). However, no significant effect was observed between two administrations in terms of negative rate of PCR on Day 14 (RR: 0.99; 95% CI: 0.55–1.80; *p *= .99), PCR negative conversion time (MD: 2.21; 95% CI: −0.13 to 4.54; *p *= .06), rate of improvement on chest CT on Day 7 (RR: 1.05; 95% CI: 0.20–5.50; *p *= .96), and hospital stay (MD: 1.51; 95% CI: −3.94 to 6.97; *p *= .59; Table 2).

#### 3.2.7 Arbidol and interferon

The meta‐analysis result showed no significant difference between exclusive arbidol and interferon/arbidol combination regarding the PCR negative conversion time (MD: −0.99; 95% CI: −16.67 to 14.69; *p *= .90; Table 2). Also, interferon/arbidol combination showed no beneficial effect compared with interferon alone regarding PCR negative conversion time (MD: 2.31; 95% CI: −7.78 to 12.40; *p *= .65; Table 2).

#### 3.2.8 Arbidol combined with traditional Chinese medicines

Several studies^52^, ^53^, ^54^ compared the efficacy of arbidol as a combination therapy with traditional Chinese medicines. The meta‐analysis of improvement rate of chest CT found no greater benefit of arbidol combined with Lianhuaqingwen compared to arbidol alone in the treatment of COVID‐19 patients (RR: 1.27; 95% CI: 0.88–1.85; *p *= .20; Table 2). Fang et al.^53^ found that the simultaneous treatment of arbidol and Lianhuaqingwen was associated with higher improvement in patients with moderate COVID‐19 compared with Lianhuaqingwen alone. There are other studies^52^, ^54^ that reported the efficacy and safety of Shufeng Jiedu capsule combined with arbidol versus arbidol alone in patients with COVID‐19.

#### 3.2.9 Sensitivity analysis

We conducted a sensitivity analysis by including the case‐series study^55^ (Table 2).

## 4 DISCUSSION

This study aimed to provide the latest available evidence on the efficacy and safety of arbidol in the treatment of COVID‐19 disease. The meta‐analysis results showed that arbidol had no clinical efficacy for all primary and secondary outcomes, including the negative rate of PCR, PCR negative conversion time, rate of improvement on chest CT, cough alleviation, hospital stay, and disease progression.

Similar to our finding, a meta‐analysis by Huang et al.^56^ indicated that arbidol was not associated with significant improvement in terms of efficacy outcomes but for the negative rate of PCR on Day 14 compared to the control group. However, they performed a subgroup analysis only on primary outcomes based on without or with antiviral drugs.

In another similar meta‐analysis done by Li et al.,^27^ arbidol was associated with a higher negative rate of PCR compared with control in patients with COVID‐19. Nevertheless, this study found no efficacy for PCR negative conversion time and improvement rate on chest CT and progression disease. The finding of these meta‐analyses for the negative rate of PCR contrast with our findings due to the differences in control groups. In fact, the present study boasts specified control subgroups in which each non‐arabidol treatment was considered as a separate control group, but other studies take more general categories into accounts such as all non‐arabidol treatments in Huang et al.'s study and all other antiviral/no antiviral drugs in the one done by Li et al. It should be noted that the inclusion of different interventions in a control group in the meta‐analysis may cause problems including the risk of bias, heterogeneity, and imprecision, which finally affect the interpretation of findings.^57^

Although the present study found no significant treatment benefit for arbidol compared with non‐antiviral interventions, recent findings from two studies^5^, ^58^ have suggested its efficacy and safety for prophylaxis in patients with COVID‐19. The result of a clinical and laboratory data analysis also^58^ showed that arbidol improved SARS‐CoV‐2 infection though without any effect on the hospitalization rate. Zhang et al.^5^ found that arbidol was associated with the improvement in SARS‐CoV‐2 infection. It seems that more evidence is needed to approve the potential of arbidol for prophylaxis of COVID‐19.

Based on the meta‐analysis results, arabidol showed different efficacies in various outcomes in comparison to other treatments. Arbidol was not more effective than chloroquine in the negative rate of PCR and PCR negative conversion time. Also, chloroquine led to a shorter length of hospital stay than arabidol. However, arbidol showed better efficacy than oseltamivir in terms of the negative rate of PCR, the length of hospital stay, and the mortality rate. Compared with lopinavir/ritonavir, arbidol had better efficacy in the negative rate of PCR and PCR negative conversion time, and also was associated with fewer adverse events, with no significant difference between them for other efficacy outcomes.

Our meta‐analysis showed that adding arbidol to lopinavir/ritonavir increased the negative rate of PCR on Day 7 and decreased PCR negative conversion time compared to lopinavir/ritonavir alone. Furthermore, simultaneous prescription of arbidol with interferon has no effect on the PCR negative conversion time in patients. Similar results were also found for interferon as a combination therapy with arbidol. The present meta‐analysis found no benefit for arbidol in combination with traditional Chines medicine. However, more studies are needed to approve this therapeutic alternative. The meta‐analysis of Huang et al.^56^ found no significant adverse events for arbidol. However, in our study arbidol was associated with higher adverse events in patients.

## 5 LIMITATIONS

Despite the efforts to minimize limitations, there were still several limitations to the current study. One of the challenging limitations of our study was the study design. Studies conducted were mostly retrospective and associated with a higher risk of bias. To reduce bias, we applied some strategies recommended by Almeida et al.^59^ Another important limitation was the location of the studies. The majority of studies were conducted in China, which makes our findings prone to a selection bias. Finally, we could not perform subgroup analyses on variables such as the severity of illness, dosage, sample size, and other variables due to an insufficient number of available studies.

## 6 CONCLUSION

The finding of this meta‐analysis revealed that arbidol was not superior to non‐antiviral treatment in patients with COVID‐19. Compared with lopinavir/ritonavir, arbidol showed better efficacy for primary outcomes. No remarkable treatment effect was observed compared with other therapeutic agents. A well‐designed randomized controlled trial with a large sample size is necessary to conclude the efficacy and safety of arbidol against COVID‐19.

## CONFLICT OF INTERESTS

The authors declare that there are no conflict of interests.

## AUTHOR CONTRIBUTIONS

*Study concept and design*: Bahman Amani and Behnam Amani. *Literature searching*: Bahman Amani and Behnam Amani. *Study selection and appraisal*: Behnam Amani and Mahsa Zareei. *Data extraction*: Sara Zareei and Mahsa Zareei. *Data analysis and interpretation*: Bahman Amani and Behnam Amani. *Critical revision of the manuscript*: Sara Zareei. All authors reviewed and edited the manuscript and approved the final version of the manuscript.

## Supporting information

Supplementary information.

## Data Availability

The data used to support the findings of this study are included within the Supporting Information file.
