# Supplementary material for: Efficacy and safety of arbidol (umifenovir) in patients with COVID‐19: A systematic review and meta‐analysis
Source: Immun Inflamm Dis. 2021 Aug 4;9(4):1197–208. doi: 10.1002/iid3.502 (PMC8426686; doi:10.1002/iid3.502)
Supplement: Supplementary file 1 — Supplementary information. [file IID3-9-1197-s001.docx]

A. Negative rate of PCR on day 14


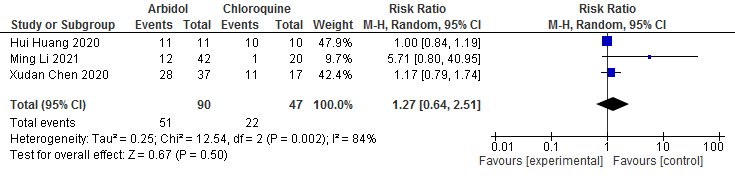


B. Hospital stay


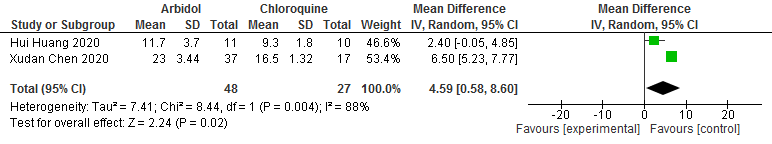


C. PCR negative conversion time


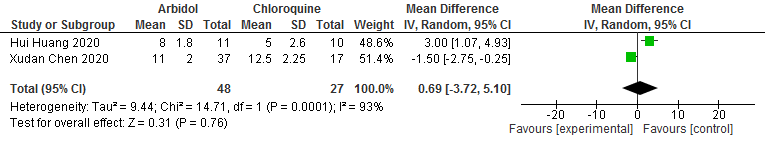


Figure 5 Forest plot of arbidol vs. chloroquine for outcomes of negative rate of PCR on day 14 (A), hospital stay (B), PCR negative conversion time (C)

A. Negative rate of PCR on day 7


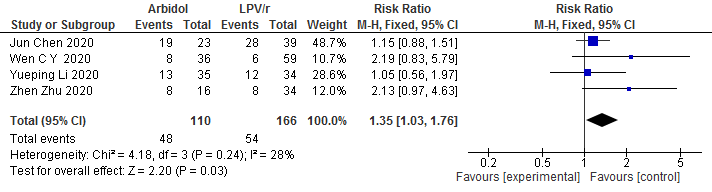


B. Negative rate of PCR on day 14


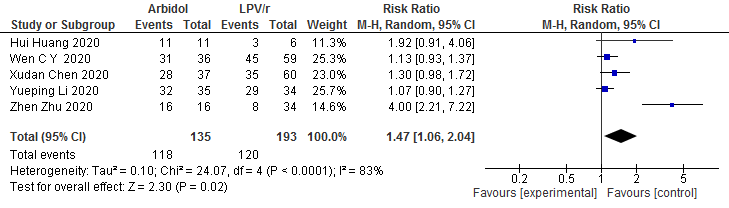


C. PCR negative conversion time


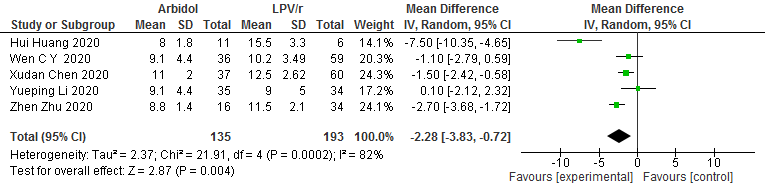


D. Rate of improvement on chest CT on day 7


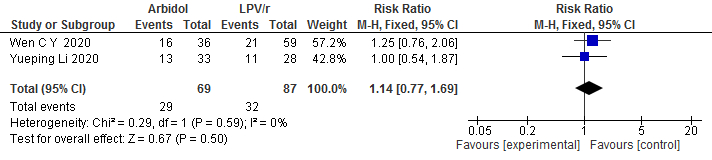


E. Rate of improvement on chest CT on day 14


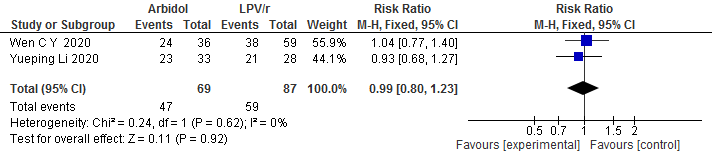


F. Rate of cough alleviation on day 7


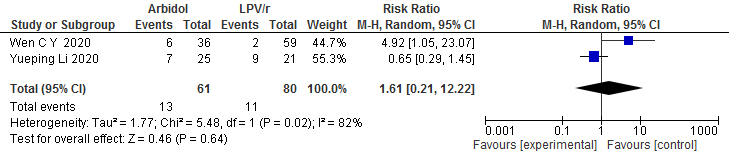


G. Rate of cough alleviation on day 14


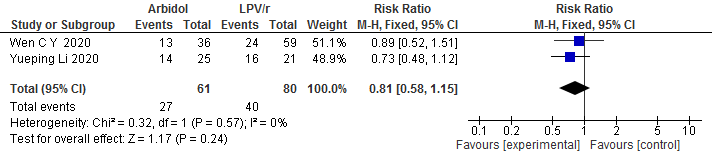


H. Hospital stay


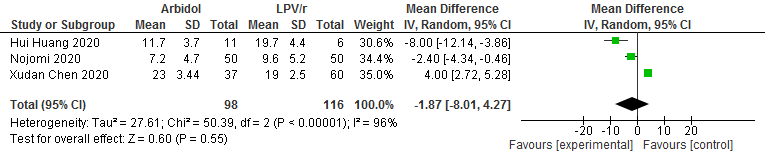


I. Disease progression


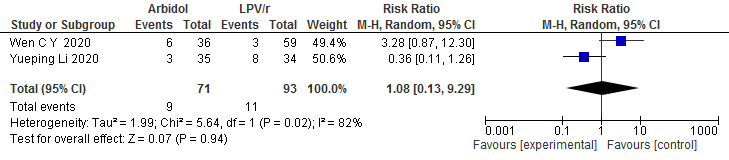


J. Adverse events


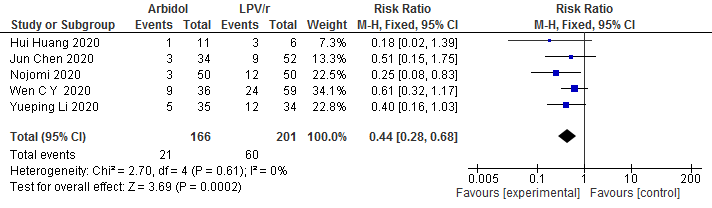


Figure 6 Forest plot of arbidol vs. lopinavir/ritonavir for outcomes of negative rate of PCR on day 7 (A), negative rate of PCR on day 14 (B), PCR negative conversion time (C), rate of improvement on chest CT on day 7 (D), rate of improvement on chest CT on day 14 (E), rate of cough alleviation on day 7 (F), rate of cough alleviation on day 14 (G), hospital stay (H), disease progression (I), adverse events (I)

A. Negative rate of PCR on day 7


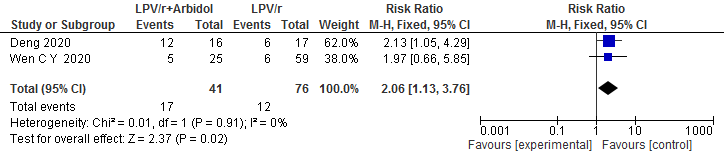


B. Negative rate of PCR on day 14


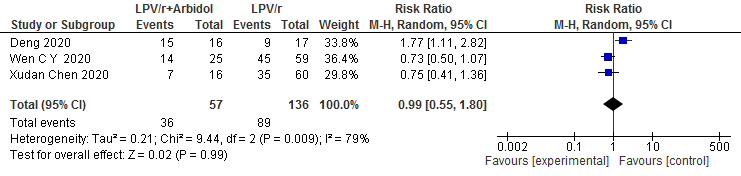


C. Rate of improvement on chest CT on day 7


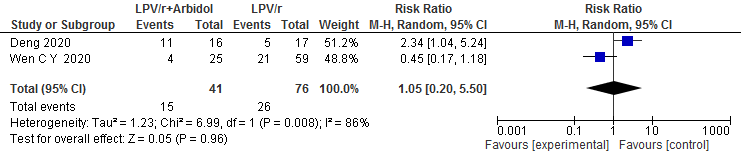


D. Hospital stay


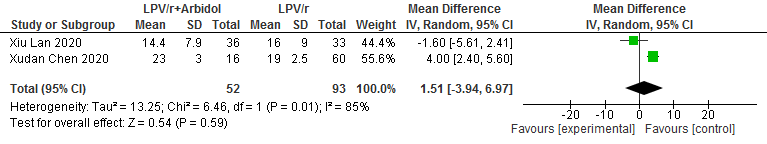


E. PCR negative conversion time


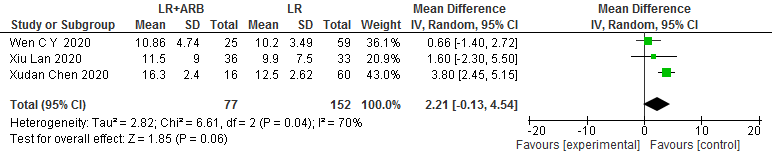


Figure 7 Forest plot of arbidol+ lopinavir/ritonavir vs. lopinavir/ritonavir for outcomes of negative rate of PCR on day 7 (A), negative rate of PCR on day 14 (B), rate of improvement on chest CT on day 7 (C), hospital stay (D)


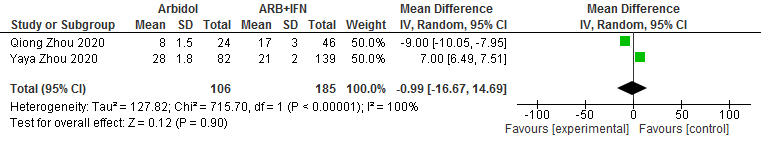


Figure 8 Forest plot of arbidol vs. arbidol+IFN for outcome of PCR negative conversion time (A)


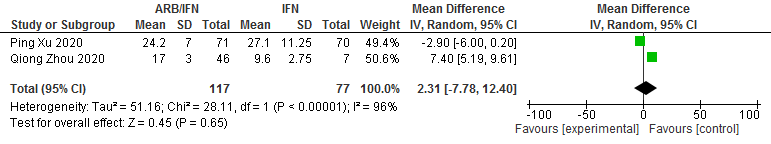


Figure 9 Forest plot of arbidol+IFN vs. IFN for outcome of PCR negative conversion time (A)


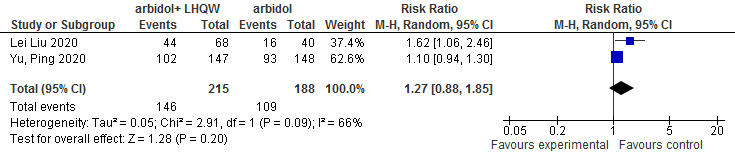


Figure 10 Forest plot of arbidol+ Lianhuaqingwen vs. arbidol for outcome of Rate of improvement on chest CT
